# Low Birthweight as a Risk Factor for Non-communicable Diseases in Adults

**DOI:** 10.3389/fmed.2021.793990

**Published:** 2022-01-06

**Authors:** Maria Eugenia Bianchi, Jaime M. Restrepo

**Affiliations:** ^1^Laboratory Physiology, Department Basic Sciences, Institute School of Medicine, National Northeast University, Corrientes, Argentina; ^2^Department of Pediatrics, Pediatric Nephrology Service, Icesi University, Valle del Lili, Cali, Colombia; ^3^Fundación Valle del Lili, Cali, Colombia

**Keywords:** LBW (low birth weight), non-communicable diseases (NCDs) and risk factors, Barker hypothesis, CKD (chronic kidney disease), glomerular number

## Abstract

According to studies undertaken over the past 40 years, low birthweight (LBW) is not only a significant predictor of perinatal death and morbidity, but also increases the risk of chronic non-communicable diseases (NCDs) in adulthood. The purpose of this paper is to summarize the research on LBW as a risk factor for NCDs in adults. The Barker hypothesis was based on the finding that adults with an LBW or an unhealthy intrauterine environment, as well as a rapid catch-up, die due to NCDs. Over the last few decades, terminology such as thrifty genes, fetal programming, developmental origins of health and disease (DOHaD), and epigenetic factors have been coined. The most common NCDs include cardiovascular disease, diabetes mellitus type 2 (DMT2), hypertension (HT), dyslipidemia, proteinuria, and chronic kidney disease (CKD). Studies in mothers who experienced famine and those that solely reported birth weight as a risk factor for mortality support the concept. Although the etiology of NCD is unknown, Barry Brenner explained the notion of a low glomerular number (nGlom) in LBW children, followed by the progression to hyperfiltration as the physiopathologic etiology of HT and CKD in adults based on Guyton's renal physiology work. Autopsies of several ethnic groups have revealed anatomopathologic evidence in fetuses and adult kidneys. Because of the renal reserve, demonstrating renal function in proportion to renal volume *in vivo* is more difficult in adults. The greatest impact of these theories can be seen in pediatrics and obstetrics practice.

## Introduction

Low weight and height newborns (NBs) (either preterm or with intrauterine growth restriction) are currently considered a public health issue. Every year, 1.1 million NBs die as a result of preterm delivery problems (1). Low birthweight (LBW) is not only a significant predictor of perinatal mortality and morbidity, but also raises the risk of chronic non-communicable diseases (NCDs), such as diabetes and cardiovascular disease, occurring in adulthood, according to studies conducted over the last 40 years (2).

LBW was identified as a future risk factor for NCDs in adults by David Barker (June 1938- August 2013), an English epidemiologist (3). To do so, he looked at the most common causes of death and their link to birthweight in a sample of 5,543 men born in Hertfordshire, England between 1911 and 1930, a population that transitioned from famine to being a pillar of industrial growth. Years later, based on studies in a Norwegian cohort population (4), he claimed that “children who develop coronary heart disease and type 2 diabetes grow slowly throughout fetal life and infancy but rapidly increase their body mass indices afterward.” Therefore, the risk factor for NCD mortality is generated not only during pregnancy, but also during the first years of life in relation to a child's nutrition, his/her environment, and associated epigenetic factors (5).

The concept of the “thrift gene” emerges for fetuses that have not been fed throughout pregnancy, as well as the hypothesis of biological plasticity: not only the genotype, but also the environment in which a life develops, is crucial. As a result, the term “fetal programming” was coined (6).

The origin of diseases associated with lifestyle is determined at the time of fertilization, passing through the stages of embryo, fetus, NB, and the first years of life, which implies the importance of the mother's health during pregnancy, according to the theory of the developmental origin of health and disease (DOHaD). Nutritional variables, as mentioned by Barker, can affect the mother's and child's health, but other factors, such as ambient air pollution (7), infectious diseases, stress, and toxins can also have an impact (8).

Barker's idea is thus a philosophical vision with substantial epidemiological foundations. It is framed in evolutionary theories, relating them to those of the French biologist Jean- Baptiste Lamark (1744-1829), who proposed that human plasticity, like that of all living beings, is influenced by epigenetic variables, as well as Darwin's natural selection rules (9).

## Adults With Lbw Die From a Variety of Causes and Previously Compared to Normal Birthweight Infants

The so-called Barker hypothesis has been established in a number of closed communities over the last 40 years with regard to NCD mortality. The requirement for obtaining this proof is birthweight records, which, according to the WHO, are not documented in even 10% of all children born. In general, this project relates to industrialized countries, where the frequency of this condition is decreasing, highlighting the necessity of such registries in undeveloped nations where the prevalence is higher (10). Norwegian studies, for example, are very strong and based on the Norwegian Medical Birth Registry, which has been recording birthweights across the country since 1967, and the Norwegian Patient Registry, which has been recording diagnostic codes for all admissions and office visits since 2008 (11). Researchers are now looking for alternative data sources, such as records from indigenous communities in Australia, which have been kept since 1956 by a Catholic mission (12).

Cardiovascular etiology, arterial hypertension, diabetes mellitus, cardiometabolic syndrome (13), and stroke were among the causes of death documented in these adult populations (14). Wendy Hoy et al. added respiratory and infectious causes of mortality in a small cohort of aboriginal adults in Australia (12).

Some characteristics of these studies can be summarized as follows: (a) In most articles, the deaths of LBW children are compared to the mortality of normal birthweight children in some cross-sectional studies. There are no control groups available. (b) The questions that arise are how old or how long it takes for LBW children to develop NCDs, as well as whether there are any other epigenetic variables in their lives that we can prevent. (c) Another factor to consider is that, because these studies were conducted on small, closed populations and life expectancy is anticipated for a given country, life expectancy for the years in which individuals were born was not included as a variable to be considered.

## Ncd in Adults Born With Lbw

Two large groups studies are identified.

a.) *Population studies related to intrauterine hunger*: The most well-known studies are those from the Netherlands (the Dutch famine) (15), Biafra (16), Austria (17), China (18), and Nigeria (19), which generally associate intrauterine hunger with the development of cardiometabolic syndrome in adulthood, in some cases showing that the risk factor (intrauterine hunger) was independent of the children's birth weight (20).
b.) *Population studies in which factors other than birthweight are not taken into account*.

Premature delivery, low birthweight, and being small for one's gestational age have all been linked to NCD in adulthood, according to a meta-analysis published recently. The following NCDs were included in the selection criteria for the systematic review stage: obesity, being overweight, adipose tissue, diabetes, insulin resistance, hyperglycemia, glucose intolerance, and metabolic syndrome. Of 8,580 articles, 28 met the inclusion criteria with sufficient data and were retrospective and prospective. Seventy-five percent included both sexes, while the others only contained men (12.5%) or women (12.5%) (21).

Another meta-analysis contains studies in which children were categorized based on their birthweight, which ranged from <2 to 4.5 kg; they have a higher risk of developing type 2 DBT, cardiovascular disease, and HT as their weight declines. They found that women are more likely to develop HT, and that the development of HT follows a J-shaped pattern (it is observed in LBW and children with high birthweight) (22).

The 1966 Northern Finland Birth Cohort examined the association between birthweight and blood pressure at 31 years old, and revealed that birthweight was inversely related to blood pressure, notably in men (23). A 1986 study of the Northern Finnish birth cohort found a similar rise in systolic blood pressure at age 16, particularly among girls (24).

In the Chinese population, through the Shanghai Women's Health Study (SWHS) and the Shanghai Men's Health Study (SMHS), which recruited more than 100,000 people, it was shown that LBW is an important risk factor for the development of obesity, diabetes, and hypertension in adulthood (25).

More than 150,000 women were enrolled in the Nurses' Health Study II (NHS II), which demonstrated that women with LBW have a higher risk of hypertension (26). The Bogalusa heart study also found that low birthweight is linked to systolic blood pressure fluctuation (27).

The limitations of these studies were: a) the researchers obtained significant results after adjusting for body size; b) various lifestyle factors—such as physical activity, smoking status, alcohol intake, family history, and socioeconomic status—were not considered in the evaluation of these relationships. Furthermore, in all populations around the world, females and males have different birthweights, so the association between birthweight and adult disease must be assessed separately for females and males (28).

## Possible Causes for the Appearance of NCDs in adults

On the one hand, the paradigmatic shift that Barker's concept entailed aimed to determine the causes of death, while on the other hand, the mechanisms by which epigenetic variables could increase the risk of HT, DMT2, and obesity in adults. The following can be mentioned:

1. Hormonal effects of the hypothalamic-pituitary axis, glucocorticoids, and growth hormone (29) act on the cardiovascular system or predispose patients to the development of obesity (8).
2. Epigenetics indicates something that we do not have in our genes, but which we can still pass on to our children. Epigenetics deals with changes in gene expression not resulting directly from mutations of DNA sequences, which lead to the formation of inherited traits both intra-generationally and inter-generationally. (30). Altered ketogenesis could be involved in the pathogenesis of DOHaD (31) through post-translational modification (32).
3. Studies that compare and contrast genetic and epigenetic effects. Mendelian randomization appears to be a new tool used in studies. The Nord-Trondelag Health (HUNT) Study is a population study that began in 1984 and involves the voluntary participation of individuals in clinical, phenotypic, and genotypic investigations (33, 34). There are Australian-Norwegian-British studies that caution about the interpretation of results deriving from Mendelian randomization (35).
4. Number of operational units: Reduced number of nephrons. Regarding the molecular mechanisms of nephron number reduction, the authors should mention oxidative stress, alterations in the renin-angiotensin system, alterations in sodium transporters, renal sympathetic activity, and the glucocorticoid effect.

Brenner et al. proposed in the 1980s (36) that in utero growth restriction results in a low nephron number, which may predispose patients to HT and kidney disease through mechanisms such as increased single nephron glomerular filtration, compensatory nephron hypertrophy, and decreased functional reserve. Approximately 60% of nephrons grow during the third trimester of pregnancy, and kidney development stops between 35 and 36 weeks of pregnancy (37). Thus, preterm delivery or delayed intrauterine growth may have a significant impact on nephron formation and nephron number (38).

Based on Guyton's basic science, physiology, and a positivist perspective, Barry Brenner and Valerie Luyck compiled reports on the consequences of LBW in the development of chronic kidney disease (CKD) (39–42).

The National Institute of Nephrology, the National Referral Center for Pathology, from Havana, Cuba, under the authorship of Reinaldo Maalich et al. demonstrated that the number of nephrons was decreased in dead fetuses, which was a paradigmatic leap from Brenner's hypothesis (43).

The lower number of nephrons would not only respond to nutritional aspects during pregnancy, but also highlight other causes, most of which mainly affect disadvantaged populations: pre-eclampsia, diabetes in gestation, maternal overweight/obesity, maternal underweight, advanced maternal age, adolescent pregnancy, and assisted reproduction. malaria during pregnancy, maternal chronic illness, and childhood overweight (44).

We agree that historical pathologic kidney descriptions identified the loss of nephrons as a cause of CKD (45). In post-mortem studies, it has been observed that total nephron number varies up to 13-fold (46). Wendy Hoy et al. showed in human kidneys, from a series of multiracial autopsies, that with age, the glomerular number decreased, and within a patient, there was a five-fold variability in mean glomerular capacity and significant heterogeneity in individual glomerular sizes. Lower glomerular number, higher body size, hypertension, and being black were all linked to larger mean glomerular volume and increased variability of individual glomerular volume. Glomerular enlargement, intimal thickening, and higher rates of glomerulosclerosis were all signs of hypertension as people became older. In whites and aborigines, but not in blacks in the US, a lower glomerular number was associated with hypertension (47).

Other studies were carried out by comparing the results of biopsies from living kidney donors associated with angiograms in the search for techniques that indicate glomerular number (48).

Imaging studies measuring renal volume were accepted to demonstrate the glomerular number *in vivo*, reverting to Dinkel's formula (49).

The most accessible were those of ultrasound, which gave rise to studies that entailed kidney measurements according to age and sex in each month and year in pediatrics (50), although not always specifying in the study sample the variables that would refer to nutritional status, blood pressure, and proteinuria of the healthy children (51, 52).

To determine kidney volume, magnetic resonance imaging (MRI) investigations are more accurate. These study lines were developed as a result of the experiences of polycystic kidney research organizations (53). Recently, a technique for measuring *in vivo* was developed, which will once again open areas of inquiry into the demonstration of Brenner's theory, such as knowing the metabolism of a fetus' cells, despite all the constraints (54).

Renal volume, as gauged by the number of nephrons, and the existence of hypertension with glomerulomegaly have already been demonstrated, but due to the kidneys' renal reserve capacity, it is much more difficult to link renal size or nephron mass (55) to renal function. New study avenues were opened to explain why renal failure or arterial hypertension were not found in persons with a single kidney, as well as in transplant donors with fewer nephrons. This disease would not arise in people with single kidneys, whether transplanted or living donors, because nephron loss must occur during a key phase (when nephrogenesis is underway), implying that the quality of remaining nephrons is more essential than the quantity (11).

In a recent survey, 2,663,010 people were involved, and after a mean follow-up of 26 years (maximum 50 years), the odds ratio (OR) for CKD was 1.72 (95% CI, 1.60 −1.90), SGA was 1.79 (95% CI, 1.65–1.94), and preterm delivery was 1.48 (95% CI, 1.65–1.94). Analyses that used CKD diagnosis at stages 3–5 as an end point yielded similar results (56).

Recently, a population-based study (not a closed one) in Australia, with a total of 4,502 individuals who reported information about their birthweight, showed that birthweight had a positive association with eGFR (28).

Therefore, the Low Birthweight and Nephron Number Working Group has recommended that people with LBW be examined and followed up with to detect kidney disease or risk factors for renal illness at a young age (57).

## Impact of LBW in Pediatrics and Obstetrics

Based on these convincing works, the care of mothers and children during the first years of life has impacted political decisions around the world (58).

Each year, an estimated 13.7 million full-term children with LBW are born around the world, accounting for 11% of all births in poor nations. These children account for 75% of NB mortalities in Latin America (59).

Caroline Abitbol et al. in Miami found a link between the appearance of proteinuria and its subsequent increase, as measured by the protein/creatinine ratio in urine, and a subsequent deterioration of glomerular filtration (*r* = 0.8, *p* = 0.0001) and a tendency toward obesity with a body mass index (BMI) greater than the 85th percentile (89 percent sensitivity, PPV: 67%, *p* = 0.03) in newborns under 1,000 g. A subsequent study by the same center looked at the effects of obesity and prematurity on renal disease progression in a retrospective cohort of these children (44 obese and 36 non-obese). When compared to obese children born at term, patients who became obese after being born extremely preterm had a higher risk of impaired kidney function during childhood (hazard ratio 2.4; 95% CI: 1.1–7.1; *p* = 0.04) (60).

The foregoing becomes one of the main objectives in the post-natal monitoring of this group of children, as it is to monitor adequate weight gain during their weight-bearing growth to avoid excess protein intake, and therefore limit secondary hyperfiltration (61).

In a striking work done in Romania, they explored whether patterns of catch-up growth affect metabolic and cardiovascular outcomes in previously institutionalized adolescents. Children who are placed for adoption have higher levels of inflammation and HbA1c than those who remain in institutions. They emphasize in the discussion that adoptive parents should be informed about the potential consequences of shifting from perinatal restricted growth and rapid weight gain in infancy, plan a balanced diet with adequate nutrition and limiting obesogenic foods, plan regular physical activities, and begin these preventive measures with their children as soon as possible (62). They do not include qualitative variables such as less stress in their new homes.

## Conclusions

The Barker hypothesis has strong connotations in the field of epidemiology from an evolutionary and mechanistic basis. The Brenner hypothesis is rooted in physiology and is positivistic. Thus, the adult patient whom we receive in the office with diabetes mellitus, obesity, HT, proteinuria, or CKD is the result of genetic and epigenetic conditions.

Maternal risks, such as overnutrition or undernutrition and GDM, which lead to low birthweight or high birthweight, certainly increase the risk for obesity and hypertension later in life as NCDs. Interventions in pre-conception or pre-pregnancy, as healthy habits, could be extremely important to reduce risks during pregnancy and the outcomes of childhood. Additionally, rapid changes in the environment, such as rapid urbanization, migration and new lifestyles, put these populations at higher risk of developing NCDs, especially in low- and middle-income countries.

In accordance with the DOHaD concept, adaptations occurring after environmental alterations would be advantageous in terms of population survival, when these alterations are consistent over several generations. However, they must be handled properly from a prevention and follow-up point of view.

## Author Contributions

Both authors listed have made a substantial, direct, and intellectual contribution to the work and approved it for publication.

## Conflict of Interest

The authors declare that the research was conducted in the absence of any commercial or financial relationships that could be construed as a potential conflict of interest.

## Publisher's Note

All claims expressed in this article are solely those of the authors and do not necessarily represent those of their affiliated organizations, or those of the publisher, the editors and the reviewers. Any product that may be evaluated in this article, or claim that may be made by its manufacturer, is not guaranteed or endorsed by the publisher.
